# Sex‐ and state‐dependent covariation of risk‐averse and escape behavior in a widespread lizard

**DOI:** 10.1002/ece3.10723

**Published:** 2023-12-11

**Authors:** Qiang Wu, Alexis Rutschmann, Donald B. Miles, Murielle Richard, Jean Clobert

**Affiliations:** ^1^ Station d'Ecologie Théorique et Expérimentale, UAR 2029, CNRS Moulis France; ^2^ MOE Key Laboratory of Biosystems Homeostasis & Protection, College of Life Sciences Zhejiang University Hangzhou China; ^3^ School of Biological Sciences University of Auckland Auckland New Zealand; ^4^ Department of Biological Sciences Ohio University Athens Ohio USA

**Keywords:** antipredator response, behavioral syndrome, gravidity, inter‐sexual difference, parasitism, state dependence

## Abstract

Mounting evidence has shown that personality and behavioral syndromes have a substantial influence on interspecific interactions and individual fitness. However, the stability of covariation among multiple behavioral traits involved in antipredator responses has seldom been tested. Here, we investigate whether sex, gravidity, and parasite infestations influence the covariation between risk aversion (hiding time within a refuge) and escape response (immobility, escape distance) using a viviparous lizard, *Zootoca vivipara*, as a model system. Our results demonstrated a correlation between risk‐averse and escape behavior at the among‐individual level, but only in gravid females. We found no significant correlations in either males or neonates. A striking result was the loss of association in postparturition females. This suggests that the “risk‐averse – escape” syndrome is ephemeral and only emerges in response to constraints on locomotion driven by reproductive burden. Moreover, parasites have the potential to dissociate the correlations between risk aversion and escape response in gravid females, yet the causal chain requires further examination. Overall, our findings provide evidence of differences in the association between behaviors within the lifetime of an individual and indicate that individual states, sex, and life stages can together influence the stability of behavioral syndromes.

## Graphical abstract

The correlation of behaviors involved in an antipredator response is of paramount ecological importance, as premature flight or prolonged duration of remaining in a refuge can result in a loss of potential opportunities to increase fitness. Here, using common lizards (*Zootoca vivipara*), we found that the “risk‐taking – escape” syndrome is contingent on sex, reproductive state, and parasite infestation, suggesting that individuals with different characteristics or life stages may choose specific combinations of behavioral traits as escape tactics. Our results may, to some extent, provide explanations for optimal escape theory and emphasize the importance of shifts in individual states in the variability of behavioral syndrome.

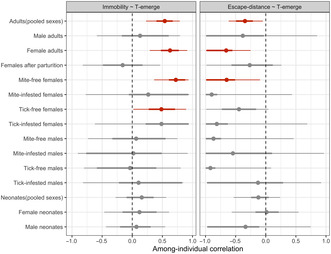

## 1 INTRODUCTION

It is established that consistent differences in behavioral traits (i.e., animal personality) can affect individual fitness (Moiron et al., 2020; Munson et al., 2020), population dynamics (Dall et al., 2012), and species interactions (Brehm et al., 2019). Behavioral differences among individuals are often heritable (Dingemanse et al., 2002; Dochtermann et al., 2015) and hence considered parts of an animal's strategy during its life. Among personality traits, antipredator behavior may be the most essential, as failure to escape can result in injury limiting future fitness or, even worse, in immediate death (Samia et al., 2016). Generally, an antipredator response consists of two major components: minimizing the probability of encountering a predator (risk‐averse behavior) and escaping (escape response) (Heithaus et al., 2009; Lima & Dill, 1990). Note that the risk‐averse decisions of an individual can be determined by their escape performance (e.g., sprint or cryptic capacities) if animals are aware of their ability to escape (Lima & Dill, 1990). Therefore, behavioral traits characterizing both the escape response and risk‐aversion may be correlated instead of functioning independently, forming a potential “risk‐averse and escape” behavioral syndrome. Nonetheless, such covariation of behaviors within the antipredator response remains largely unexplored (Cooper & Blumstein, 2015; see Ortiz‐Jimenez et al., 2022).

The correlation between escape response and risk‐averse behavior is of paramount ecological importance, as premature flight or prolonged duration of remaining in a refuge can result in a loss of fitness (Miles et al., 2001; Samia et al., 2016). A positive correlation between escape behavior and time spent in a refuge could lead to a reduction in fitness for individuals. In contrast, a negative correlation could suggest a possible compensation for costs because of a rapid resumption of activity (Ortiz‐Jimenez et al., 2022). Based on optimal escape theory, animals should maximize fitness by balancing the costs and benefits of fleeing (Cooper & Frederick, 2007; Ydenberg & Dill, 1986).

Given that organisms usually have different intrinsic attributes (e.g., sex) and live in changing environments, the ability to adjust behaviors, and therefore behavioral syndromes, according to individual phenotypes and specific environments would be critical to maximizing fitness (Royauté et al., 2019). For example, recent reviews have suggested that differences in life history driven by sex attributes can explain the emergence of animal personality and behavioral correlations (Biro & Stamps, 2008; Schuett et al., 2010; Wolf et al., 2007; Wolf & Weissing, 2010). An illustration is the sex‐specific expression of behavioral syndromes in a water strider species, *Gerris gracilicornis*, when exposed to predation risk (Han et al., 2015). Female and male water striders diverge in their life‐history characters and hence behavioral strategies for the sake of optimal fitness, which contributes to the formation of intersexual differences in the covariation of behavioral traits.

On the other hand, variation in personality and behavioral syndromes can also come with varying individual states, which are defined as “any features that affect the costs and benefits of behavioral actions” (Biro & Stamps, 2008; Dingemanse & Wolf, 2010; Luttbeg & Sih, 2010; Sih et al., 2015; Wolf & Weissing, 2010; but see Niemelä & Dingemanse, 2018). Life stage is a typically state‐dependent characteristic that has long been recognized to have an overwhelming effect on behavior. For example, most adult females, especially in live‐bearing species, experience an increased physical burden, which induces changes in locomotor performance, hormone levels, metabolic rates, and thermoregulation patterns (Bonnet et al., 2001; Braña, 1993; Hare & Cree, 2010; Le Galliard et al., 2003; Miles et al., 2000; Munns et al., 2015; Olsson et al., 2000). In turn, these changes can make gravid females more vulnerable to predators, and can have cascading effects on interconnected behavioral traits (Miles et al., 2007), and affect behavioral syndromes. Such changes can also eventually be transmitted to the offspring (Dall et al., 2004). Yet, few studies have quantified the behavioral syndrome for both mothers and their neonates.

Finally, another source of state‐dependent changes in behavior can be extended to the interaction with other species (Sih et al., 2015). One notable example is host–parasite interactions (Moore, 2002; Poulin, 2007). Parasites are known to manipulate host behavior directly (Thomas et al., 2005) or through indirect pathways, such as affecting host physiology or body condition (Thomas et al., 2005). In general, parasites are predicted to modify host behavior to benefit transmission to other hosts or their own replication (Barber & Dingemanse, 2010; Poulin, 2007, 2013). However, due to the multidimensional nature of parasite manipulation, coordinated changes in several behavioral traits may yield even greater benefits for parasites (Thomas et al., 2010). Therefore, linking personality and behavioral syndromes with parasite‐associated changes in host behavior can advance our understanding of parasitic manipulation and its potential consequences (Poulin, 2013; Thomas et al., 2010).

The aim of the present study was to examine the covariation of risk‐aversion and escape behavior in the common lizard *Zootoca vivipara*. Generally, adult males and females diverge in their activities and survival rates in this species. Males emerge earlier and engage in scramble competition for mating opportunities with females, resulting in lower summer survival due to depletion of energy reserves and predation exposure (Massot et al., 2011). During gestation, females experience reduced locomotor performance due to the reproductive burden (defined as the mass of the litter) and modify their thermoregulatory behavior and escape distance (Bauwens & Thoen, 1981; Le Galliard et al., 2003; Lecomte et al., 1993). After parturition, females prepare for hibernation by increasing foraging activity to replenish energetic reserves. Note that stress induced by predation risk during gravidity is also known to shape offspring's phenotypic responses (Bestion et al., 2014). Further, common lizards may be infested with different ectoparasites (Wu et al., 2019), which can in turn affect locomotor performance (Sorci et al., 1994) and the survival of an individual lizard (Wu et al., 2022). Thus, *Z. vivipara* is an ideal model species to test the stability of the “risk‐aversion and escape” behavioral syndrome across sexes, states (gravid vs. postpartum; infested vs. noninfested) and life stages (adult vs. neonate).

**FIGURE 1:**
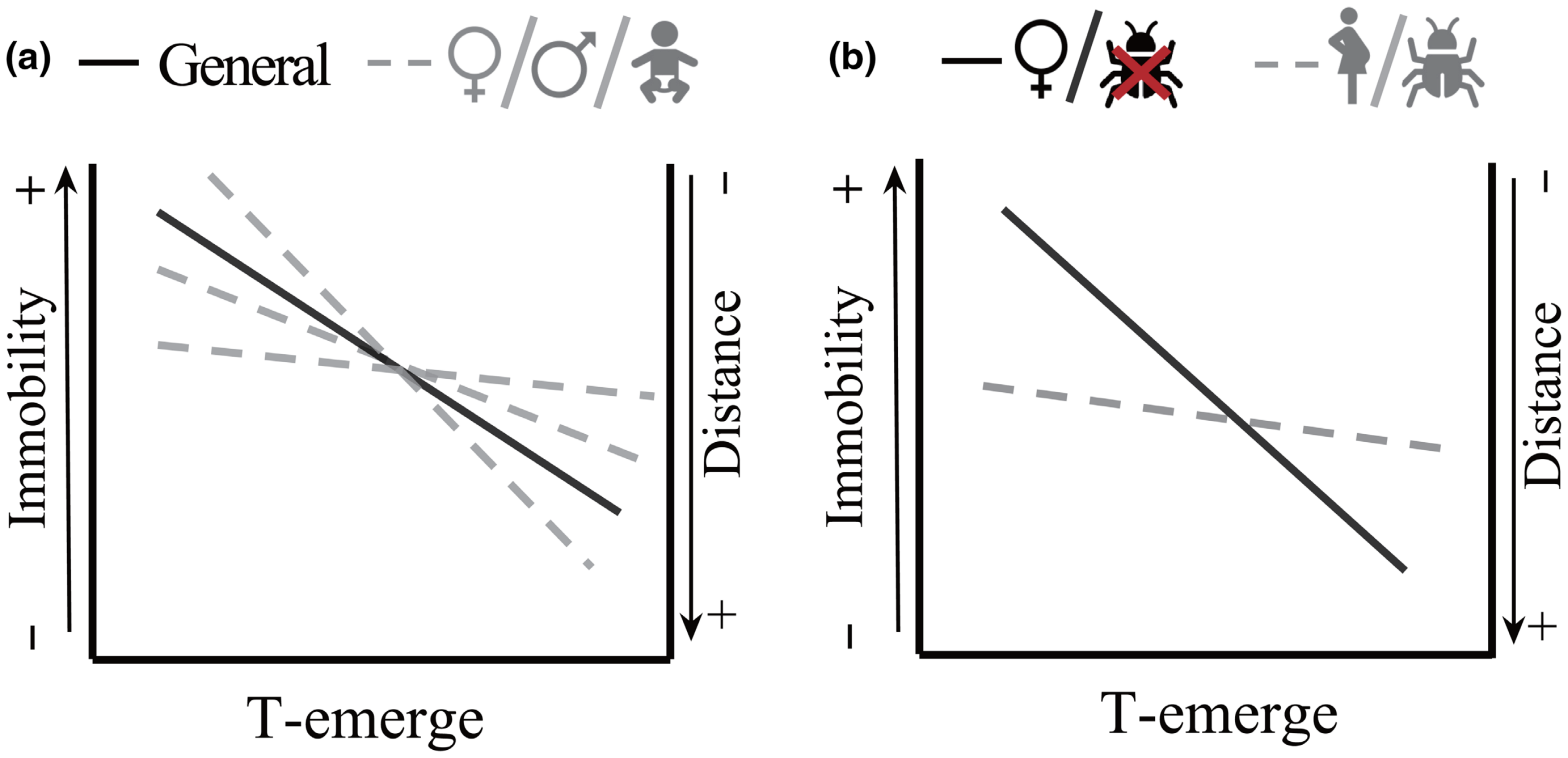
The hypothetical scheme showing how the among‐individual correlation between risk‐averse and escape behavior may differ across sexes and ages (a) and how the correlation may be affected by gravid and infestation status in common lizards (b). Note, the left and right‐vertical axes in the figure have the opposite direction.

In our study, we defined “escape behavior” in two ways: (1) the distance covered by an individual exposed to a simulated predator attack (Escape distance) and (2) the number of attacks necessary to stimulate the escape behavior (Immobility). We then quantified the “risk‐averse behavior” as (3) the time to emerge from a refuge after a simulated predator attack (T‐emerge). We characterized a “risk‐averse and escape” behavioral syndrome (hereafter “RAE syndrome”) by focusing on the correlation of T‐emerge with either Escape distance or Immobility. We predicted that (1) risk‐averse individuals will not only escape earlier (i.e., low immobility scores) and further (i.e., high distance scores), but also emerge later once attacked. As such, T‐emerge should correlate positively with Escape distance, but negatively with Immobility (Figure 1a). Also, we expect the RAE syndrome will be in the same direction (i.e., correlation sign) for neonates, males, and non‐gravid females but may vary in magnitude (Figure 1a). Second, due to the constraints on locomotor performance imposed by the reproductive burden, gravid females may have less among‐individual variation in escape response compared with males. Accordingly, we predict (2) the RAE syndrome will diminish or even reverse in gravid females (Figure 1b). This prediction is premised on the switch in predator evasion tactics during reproduction: females should shift their strategy toward a crypsis (i.e., higher Immobility and longer time to emerge), as relying on speed to avoid predation is not an adaptative option when carrying eggs (Bauwens & Thoen, 1981). Finally, we predict (3) parasite‐free individuals to present a different syndrome (either in presence or magnitude) than parasitized individuals (Figure 1b), as parasite infestation may similarly mitigate the among‐individual covariation of behavioral traits by reducing escape abilities.

## 2 MATERIALS AND METHODS

### 2.1 Study system

The common lizard *Z. vivipara* is a small, terrestrial species (adult snout‐vent length, SVL 50–70 mm) that has a broad distribution across northern Eurasia. In the Massif Central (France), females are live‐bearing, and gestation stage lasts approximately 2 months, from early June to mid‐July. After parturition, neonates emerge from a thin shell membrane within 1 h and thereafter are autonomous.

Common lizards are infested by two types of ectoparasites in our study sites (Wu et al., 2019): a hematophagous mite in the genus *Ophionyssus* (Sorci et al., 1997) and a tick, *Ixodes ricinus*. Both mites and ticks are temporary parasites that have a free‐living stage in the soil and attach to hosts for a blood meal (Cooper & Jackson, 1981). The main hosts of ticks are large mammals like deer, cattle, and sheep (Gray et al., 2009). In contrast, the primary hosts of mites are lizards.

### 2.2 Animal husbandry

We captured lizards at four sites in the Massif Central during the breeding season of 2016. The four sites are in the same mountain range at a similar elevation. At each site, we captured 4–8 males and 19–22 females (Table 1). The average sample date was June 27 (±3.5 days), depending on the local weather (Rutschmann et al., 2016). All adult lizards were transported to a field station situated in Villefort, where we measured the length (±0.5 mm) and mass (±0.01 g) of the lizards. We visually inspected lizards for the presence of ectoparasites and removed them before they entered the breeding facilities. Lizards were individually housed in a plastic terrarium (18 × 11 × 11 cm; *L* × *W* × *H*) with a layer of sterilized soil as a substrate and a cardboard shelter (4 × 10 × 3 cm; *L* × *W* × *H*) as a shelter. All lizards were reared under standardized conditions, with food and water provided *ad libitum* (Massot & Clobert, 2000). In addition, lizards were provided with a source of heat to allow for thermoregulation. After parturition, all neonates were measured and weighed. The sex of neonates was determined by counting ventral scales (Lecomte et al., 1992). Three days later, females and their neonates were released at their exact capture locations.

**TABLE 1:** Description of capture sites and sample size of each sex for adults and neonates in the Massif Central.

Site	Mountain range	Elevation (m)	Adults		Neonates	
			Male	Female	Male	Female
BOU	Margeride	1410	6	21	30	26
COM	Margeride	1435	8	21	46	29
LAJO	Margeride	1351	6	22	43	28
PAR	Margeride	1415	4	19	28	16

### 2.3 Behavioral assays

We measured risk‐aversion (A) and escape behavior (B) for both adults and lab‐born neonates. All behavioral data were collected during the lizard's natural activity period (10 am–5 pm) at the same standard temperature (27 ± 2°C). Each behavioral test was repeated three times for adult males and gravid females. Due to logistical constraints, 51% of the adults (*n* = 53) followed a fixed order of behavioral assays: B1‐A1‐B2‐A2‐B3‐A3, while 49% (*n* = 51) followed a more flexible order: either B1‐A1‐A2‐B2‐A3‐B3 or B1‐B2‐A1‐B3‐A2‐A3. The different orders were used to test the effects of assay sequences. Risk‐averse and escape behavior tests were repeated twice for post‐parturition females. Neonates were also tested twice for both risk‐averse and escape behavior with 24 h rest between behavioral assays. Due to a time constraint (neonates have to be released within 3 days after birth), we were unable to arrange different orders of behavioral tests for neonates.

#### 2.3.1 Risk‐averse behavior

To assess the risk‐averse behavior, we followed the method in Le Galliard et al. (2015). We recorded the time a lizard emerged from a refuge after simulated predation attacks (Le Galliard et al., 2015; López et al., 2005). Each lizard was individually placed in a plastic box (30 × 18 × 18 cm; *L* × *W* × *H*) equipped with an opaque plastic refuge (7 × 3 cm; Diameter × *H*) at one side and a heat source (25 W bulb) at the opposite side to provide a stimulus for basking. The wall of the box was opaque to prevent lizards from seeing their conspecifics during the trial. We filled the box with a layer of clean sand and changed the substrate for each trial. We also cleaned the terrarium and the refuge between each trial. The test started after an acclimation period of 5 min for each lizard. We then put lizards near the entrance of the refuge and simulated predation attacks with a paint brush to tap the tail until the lizard fled into the refuge (immobility from the escape behavior was used as an approximation to control for individuals' variation in response to attacks, and it showed no impacts on repeatability). If the lizard was already inside the refuge (in rare cases), we simulated several attacks at the opening of the refuge. The experimental set‐up was isolated from the observer by being placed behind sheets. We used video cameras to record the behaviors and quantify the time until emergence. We calculated the latency time for the lizard's head to emerge from the refuge (T‐emerge) to quantify the risk‐averse behavior. If an individual did not emerge after 50 min (9% of behavioral trails), we arbitrarily assign the T‐emerge as the maximal recording time. Risk‐averse behavioral tests were conducted on the 8th, 11th, and 17th days of captivity for each adult individual.

#### 2.3.2 Escape behavior

To assess escape behavior, we chased lizards down a race track (220 × 30 × 25 cm; *L* × *W* × *H*) and recorded their behavioral responses to a simulated predator attack. We first placed the focal lizard under a darkened shelter for 1 min to acclimate the individual. We then lifted the shelter and immediately simulated continuous predation attacks by stimulating individuals with a paintbrush every second, until the lizard initiated an escape response. When the lizard stopped running for more than 2 s or ran to the end of the racetrack, the trial was considered finished. We defined the number of attacks to initiate a lizard's escape as the score of “Immobility” and the final escape distance as the “Escape‐distance.” The escape behavior was tested three times for each individual on the 6th, 10th, and 16th day of captivity of adult lizards. The same experimenter conducted all escape experiments to avoid any observer effects.

### 2.4 Statistical analyses

#### 2.4.1 Repeatability and variance components

Repeatability, the measure of behavioral consistency, is often estimated by using the intra‐class correlation coefficient (Wolak et al., 2012) across different trials. However, the direct comparison between repeatability (ratios) may be misleading and may mask important information (Dingemanse et al., 2022; Royauté & Dochtermann, 2021). Here, we first calculated the repeatability (*R*) for all traits of risk‐averse and escape behavior based on the formula:

$$R=\frac{VR_{a}}{VR_{a}+VR_{w}+VR_{\text{site}}},$$

where $VR_{a}$ is the among‐individual variance, $VR_{w}$ is the within‐individual variance, and $VR_{\text{site}}$ is the among‐site variance.

Following Royauté and Dochtermann (2021), we next compared the variance components of $VR_{a}$ and $VR_{w}$ between sexes or states by exploring Bayesian linear mixed models. In the Bayesian models, we used either risk‐averse or escape behavior as the response variable while including study site and individual identity as random factors. We allowed both among‐ and within‐individual variance to vary with sex or individual state with respect to each corresponding model to test these factors. We reported the effect size (log response ratio, lnRR) to quantify the magnitude of differences in repeatability or variance components between different treatments:

$$\text{lnRR}=\ln\left(\frac{V_{x,2}}{V_{x,1}}\right)$$

where *V*_*x*,2_ can be attributed to the values of the repeatability or variance component of females, while *V*_*x*,1_ is the value of males. Similarly, when considering the effects of gravidity or infestation status, *V*_*x*,2_ represents the repeatability or variance components of gravid females or parasite‐free individuals, while *V*_*x*,1_ represents that of post‐gravid females or parasite‐infested individuals.

We extracted the posterior distributions of effect size and computed the 95% credibility interval. The Bayesian model setting and inspection of convergence have been set the same as the models for examining behavioral syndromes afterward (see details in Section 2.4.2 below).

#### 2.4.2 Behavioral syndromes

To detect potential behavioral syndromes, we partitioned phenotypic correlations into among‐individual and within‐individual correlations. To do so, we ran Bayesian multivariate generalized linear mixed models through the “MCMCglmm” package (Hadfield, 2010). The two traits of the escape behavior (Immobility and Escape‐distance) and one trait of the risk‐averse behavior (T‐emerge) were used to calculate pairwise correlations for examining potential behavioral syndromes. We specified an uninformative prior and a low degree of belief. Each model was run for 2,100,000 iterations, with a burn‐in of 100,000 and a thinning interval of 1000. We visually assessed model convergence by plotting the trace for the estimated parameters to ensure models were properly specified (Figures S1–S4). We also inspected the autocorrelations between draws, and all the autocorrelations were less than .1, as suggested by Hadfield (2012).

After decomposing the variance into among‐individual and within‐individual components, we calculated the correlation at a given level by dividing the covariance between two traits by the square root of the product of the two variances following Brommer et al. (2014):

$$\mathrm{Cor}=\frac{{\mathrm{Cov}}_{1,2}}{\sqrt{{\mathrm{Var}}_{1}\times {\mathrm{Var}}_{2}}}.$$

We used the mode of the posterior distribution as the point estimate at a given statistic and the 95% highest posterior density as the 95% credibility interval. Credibility intervals that do not overlap with zero were considered significant. We specified random intercepts for both individual identities and populations. We first ran the model for all adults with sex as a fixed effect and body condition as a covariate (the latter was calculated as the residuals from a regression of body mass against SVL). We then ran similar models for different sexes and different individual states, with body condition included as a covariate. Moreover, gravid females tend to become less mobile as they approach parturition and may bear different reproductive burdens (Le Galliard et al., 2003). Therefore, we added (1) the number of days between behavioral measurements and parturition dates and (2) size‐specific reproductive burden as covariates when examining behavioral syndromes in gravid females. We first calculated mass differences between gravid and post‐gravid females and then extracted the residuals of mass differences against SVL as a size‐specific burden for pregnant females. Finally, we calculated the differences in among‐individual correlations between different sexes or states (ΔCor_1,2_ = Cor_1_ − Cor_2_). This analysis allowed us to investigate whether the magnitude of the correlation significantly differed between different treatment groups. Similar model structures and analyses were also built for neonates following the procedures above.

The sequence of behavioral assays might have an effect on subsequent behavioral responses, which we called here “order effects,” also known as “carry‐over effects” (Dochtermann, 2010). Though often ignored in data analyses, order effects might change the magnitude of observed correlations between behavioral traits (Logue et al., 2009). Therefore, in the models testing for both repeatability and behavioral syndrome, we included the order of behavioral trails as a fixed variable to control for the potential order effects. All the statistical analyses in this study were performed in R version 4.0.2 (R Core Team, 2020).

## 3 RESULTS

### 3.1 Repeatability and variance components

**FIGURE 2:**
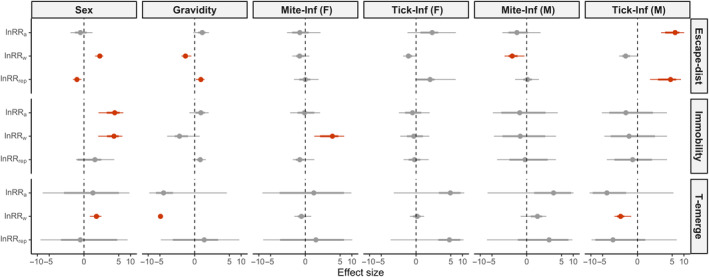
Effect sizes of different sexes and individual states in affecting the repeatability, among‐individual variance, and within‐individual variance of risk‐averse and escape behavior. Panels from left to right respectively represent the effect size of (1) Sex (females vs. males); (2) Gravidity (before vs. after‐parturition); (3) Mite infestation on females (mite‐free vs. mite‐infested females); (4) Tick infestation on females (tick‐free vs. tick‐infested females); (5) Mite infestation on males (mite‐free vs. mite‐infested males); (6) Tick infestation on males (tick‐free vs. tick‐infested males). The effect size is represented by the log response ratio (lnRR_rep_, lnRR_a_, and lnRR_w_ stand for effect sizes of treatments in affecting repeatability, among‐individual variance and within‐individual variance, respectively).

For adults, all three traits of risk‐averse and escape behavior were all significantly repeatable (Figure S5). However, the repeatability of these traits did not differ between sexes or individual states except for Escape distance (Figure 2). In fact, we found that sex and individual state mainly influenced the VR_w_ component of risk‐averse and escape behavior (Figure 2). Specifically, females had a higher VR_w_ than males in all traits of risk‐averse and escape behavior. Meanwhile, gravid females had a lower VR_w_ in Escape distance and T‐emerge compared with post‐gravid females. Similarly, males without an infestation of mites or ticks also had a lower VR_w_ than infested males in these two behavioral traits. In contrast, the behavior of gravid females seemed less impacted by parasite infestation.

For neonates, we found that female neonates had a lower VR_w_ in risk‐averse behavior (T‐emerge) compared with male neonates. The rest of behavioral traits for neonates exhibited no differences in either repeatability or variance components between the sexes (Figure S6).

### 3.2 Behavioral syndromes

**FIGURE 3:**
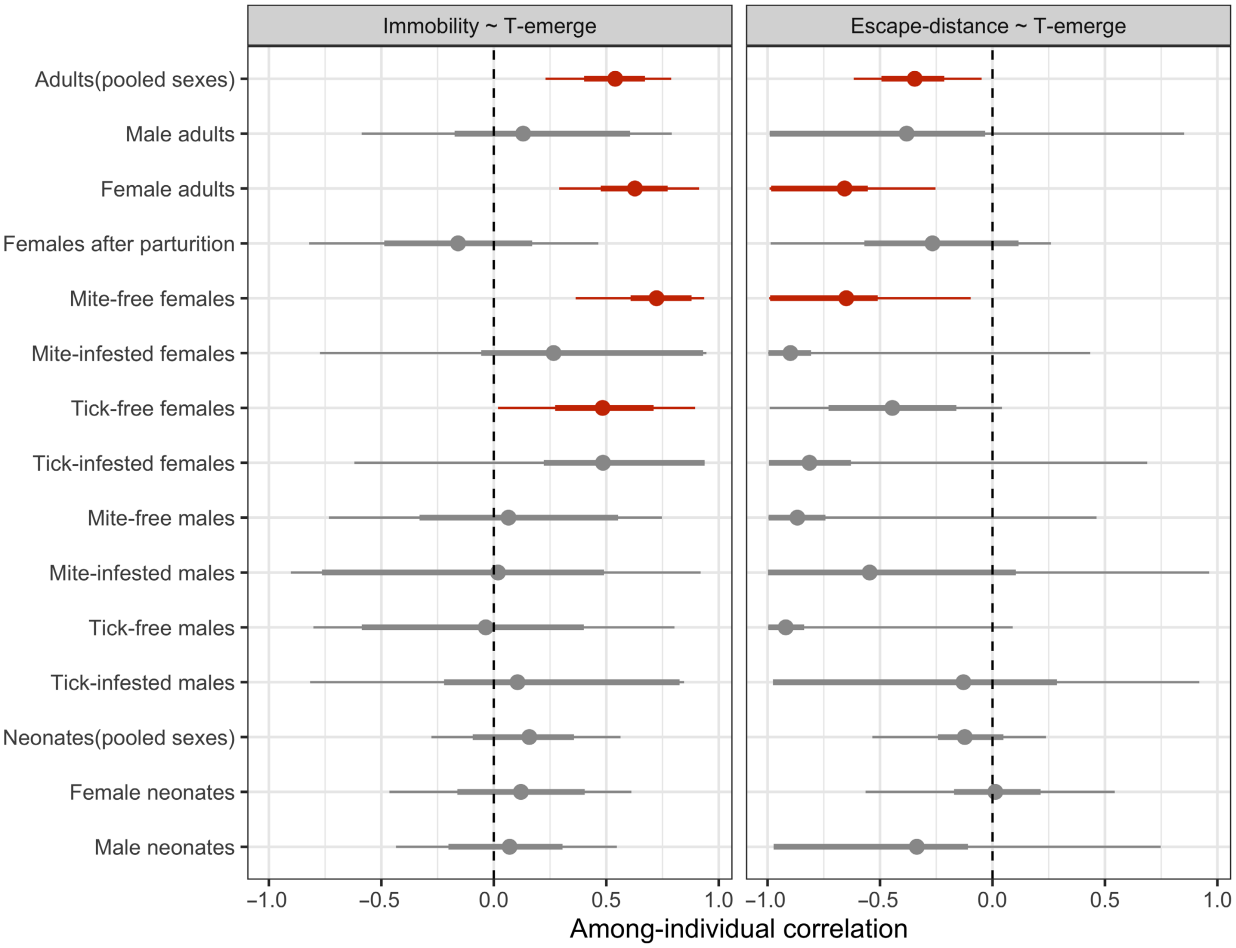
The posterior distribution of magnitude differences in among‐individual correlations of risk‐averse and escape behavior between different sexes, gravid status, and infestation status. The bolder error bars represent the 66% credibility intervals while the slim error bars the 95% credibility intervals. Error bars that do not overlap with zero indicate significant differences in correlations at each respective level.

The presence of RAE behavioral syndrome differed between the sexes and between different states of gravidity or infestation (Figure 3). When pooling adults together, we found significant correlations between Escape‐distance and T‐emerge as well as between Immobility and T‐emerge at the among‐individual level. However, when splitting data for each sex, the results indicated that the among‐individual correlations remained only in gravid females and not in males. Moreover, the RAE syndrome in gravid females vanished after parturition or when infested by either kind of parasite (nonsignificant among‐individual correlations Escape‐distance and T‐emerge and between Immobility and T‐emerge, Figure 3).

**FIGURE 4:**
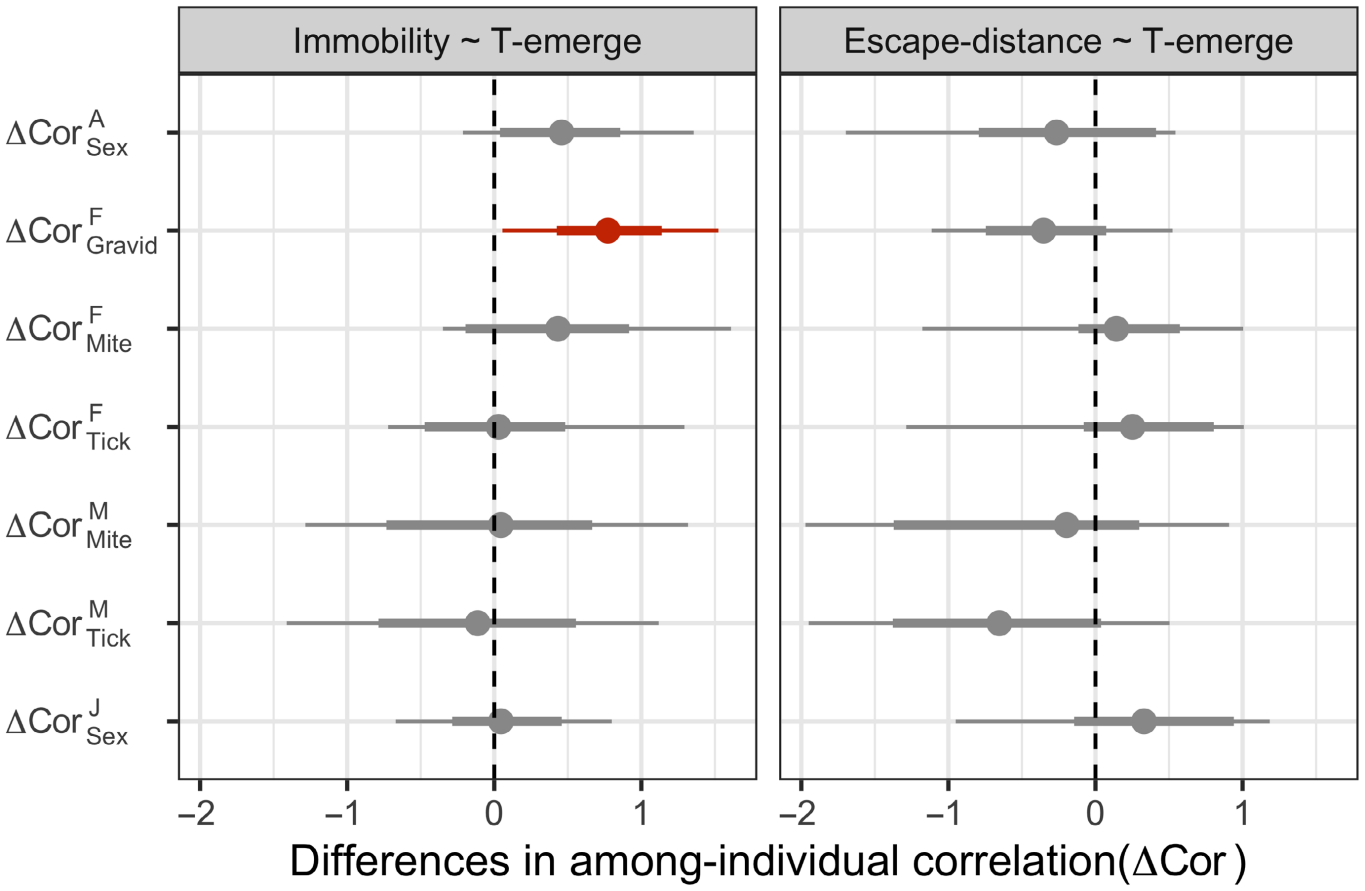
The posterior distribution of among‐individual correlation of risk‐averse and escape behavior for adults and offspring. The bolder error bars represent the 66% credibility intervals while the slim error bars the 95% credibility intervals. Error bars that do not overlap with zero indicate significant differences in correlations at each respective level.

We then compared the magnitude of RAE behavioral syndrome between the sexes and different individual states. We found no differences in the magnitude of RAE behavioral syndrome either between the sexes or between infestation status (Figure 4). However, gravidity seemed to play a role that gravid females exhibited a stronger magnitude in the correlation between Immobility and T‐emerge compared with post‐gravid females.

Finally, no significant among‐individual correlations were detected between risk averse and escape behavior when either pooling neonates overall or separated by sex (Figure 3). Therefore, we found no evidence that the RAE behavioral syndrome may emerge early in the ontogeny of common lizards.

## 4 DISCUSSION

In this study, we found variance components of risk‐averse and escape behavior to differ between sexes or individual states. We further found significant among‐individual correlations between risk averse and escape behavior, confirming the existence of a behavioral syndrome in *Z. vivipara*. Yet, the syndrome only existed in gravid females and disappeared after parturition and when being infested by parasites. Our study highlights the importance of comparing variance components in behavioral consistency and illustrates that the expression of behavioral syndromes can differ between sexes and can be mediated by individual states.

### 4.1 Consistent differences in behavior

In this study, only the repeatability of Escape distance varied across sexes and different individual states. However, complementary analyses of variance components highlighted the effects not detected by repeatability. For example, Immobility and T‐emerge showed sex‐specific differences in variance components but not in repeatability. The comparison of variance components can help to reveal the biological meaning of behaviors: a higher among‐individual variance (VR_a_) indicates that individuals are more behaviorally distinct from each other, whereas a higher within‐individual variance (VR_w_) reflects higher behavioral plasticity (Royauté & Dochtermann, 2021). Here, our results indicated that gravid females exhibited greater plasticity (a higher VR_w_) than males in all three behavioral traits, and this intersexual discrepancy in behavioral plasticity is enhanced after parturition (Figure 2). Males engage in limited seasonal variation in behavior (e.g., foraging, seeking mating), hence facing a limited variation in exposure to predation. On the other hand, females have to contend with pre‐, during‐, and post‐gestation states, each of which involves with different uses of home range and constraints on mobility. While being more explorative when not gravid (i.e., for foraging), females tend to stay close to their refuge once they experience limitations in locomotion induced by the reproductive burden (A. Rutschmann, D. Miles, personal observation). As such, females may exhibit higher behavioral flexibility to adapt to each life‐history stage, but this remains to be tested.

Furthermore, both mite‐ and tick‐infested males had a higher VR_w_ in Escape‐distance and T‐emerge compared to parasite‐free males. Given that mites and ticks are both ectoparasites that do not use *Z. vivipara* as an intermediate host to complete their life cycles (i.e., predation on hosts also means the dead end of ectoparasites), a higher behavioral plasticity may, to some extent, reduce the overall predation risks for infested individuals (Cooper & Blumstein, 2015). However, our results are inadequate to conclude whether the changes in behavioral flexibility result from parasite manipulation or from side effects correlated with infestation. Further work is needed in our system to reveal the cause‐and‐effect link.

### 4.2 Correlation between risk‐averse and escape behavior: A sex‐ and state‐dependent strategy

Our results pointed out the existence of correlations between the measured behaviors. Yet, we only found significant correlations in gravid females. As expected, we found that gravid females remaining in their refuge for a prolonged period of time also show a higher immobility (positive correlation between Immobility and T‐emerge) and a shorter escape distance (negative correlation between Immobility and T‐emerge). However, contrary to our expectations, the syndrome did not exist in neonates, males, or post‐gravid females. The expression of the syndrome therefore seems to mainly depend on the locomotor burden imposed by the litter (Le Galliard et al., 2003). As already discussed, their reproductive burden is likely to handicap gravid females and limit their foraging and escaping abilities. Further, because of reduced locomotion, movement performance is likely to be less efficient, especially when facing increased energy demand during gestation (Foucart et al., 2014). Gravid females therefore have to change their behavior to avoid predation and fulfill energy consumption.

A striking result is that behavioral syndromes are not stable throughout an individual's lifetime. First, the expression of among‐individual correlations disappeared post‐parturition. The stability of behavioral syndromes has been examined in many studies, yet mixed results were found (Sih et al., 2004). Generally, one explanation for the existence of a behavioral syndrome is that the payoffs for certain combinations of behavioral traits outweigh others (Bell & Sih, 2007; Sih et al., 2004). Here, the association between risk‐averse and escape behaviors seems to be advantageous only for females during their gravidity. After parturition, females recover their mobility, locomotor performance (Le Galliard et al., 2003), and escape capability (Cooper & Blumstein, 2015). Further, *Z. vivipara* displays no parental care, and the fate of offspring should not constrain females once eggs are laid. Consequently, females can engage in intense foraging and replenish their energy reserves to enhance overwinter survival and ensure future reproduction (Bleu et al., 2013). This suggests that, after parturition, most females return to strategies akin to those of males. It is also important to remember that escape responses consist of many other components, including crypsis, speed, maneuverability, spatial memory of the habitat, or refuge usage. Maximizing the value of all these traits is not necessary to avoid predation (Domenici et al., 2011). Thus, although we found no RAE syndrome in males, nongravid females, or juveniles, other components of the escape response could have been more important.

In addition to reproduction, we also found that parasites might impact the covariation between risk‐averse and escape behaviors. Yet, the among‐individual correlation was evident only among females without parasites (Figure 4). Barber and Dingemanse (2010) proposed that parasites may decouple behavioral syndromes by affecting only one personality axis independent of the others or by strengthening the correlation between previously uncorrelated behaviors. However, this is not the case here, because we did not find that parasites differentially affected risk‐averse and escape behavior. We suggest the disassociation of behavioral syndromes in parasitized females may not be due to manipulation by parasites but rather to the pathological effects of parasitic infestation. This hypothesis might be supported by the fact that, at the time of experimental tests, we physically and chemically removed parasites from all individuals. Accordingly, the only chronic effects of parasites can explain the observed difference. However, the nonsignificance of the correlation between behavioral traits in parasitized females might also result from the low statistical power (15 and 23 unique individuals for mite‐ and tick‐infested females, respectively).

We found no significant among‐individual correlations between risk‐averse and escape behavior in lab‐born juveniles. A similar phenomenon has also been observed in other lacertid species, like *Lacerta viridis* (Bajer et al., 2015). Antipredator response usually represents the last line of defense for evading a predator, and there are few chances for an individual to learn from conspecifics (Cooper & Blumstein, 2015). Thus, one may expect the antipredator traits and their correlations to be under tight genetic control. However, our results suggest that the RAE syndromes might develop through the process of ontogeny or emerge just based on an animal's individual experience or particular states (gravidity in our case), which is a common mechanism in nature as discussed in other studies (Bajer et al., 2015).

Finally, our study suggests several avenues for additional experimentation. First, experiments that manipulate the abiotic environment (e.g., different temperature gradients) should be considered to better explore escape response. Further, traditional measurements on risk‐averse behavior may be affected by housing stress, activity, and explorative behavior, which should be controlled in future experimental designs. In addition, mechanisms of how parasites affect behavioral syndromes requires manipulated infestations to disentangle direct and indirect effects of parasitism on the stability of behavioral syndromes study. Last, we suggest expanding the types of measurements to quantify escape behavior. We encourage behavioral ecologists to incorporate more behaviors for a better understanding of behavioral covariation in the escape process.

## 5 CONCLUSIONS

In this study, we provide insights on how different sexes and state‐dependent factors can affect personality and the stability of behavioral syndromes. In the common lizards, the among‐individual correlation between risk‐averse and escape behavior is contingent on sex, reproductive state, and parasite infestation, indicating that individuals with different characteristics or life stages may exhibit specific combinations of behavioral traits as escape tactics. The variability of a behavioral syndrome can therefore be masked if one focuses on pooled sexes or fails to consider the life stage, individuals status, or the ecological environment of focal individuals. Our findings motivate future studies to integrate multiple attributes of organisms to provide underlying mechanisms of behavioral syndromes' variability and reveal how the emergence of behavioral syndromes may impact population dynamics and species interactions.

## AUTHOR CONTRIBUTIONS

**Qiang Wu:** Conceptualization (equal); formal analysis (lead); investigation (equal); methodology (equal); visualization (lead); writing – original draft (lead). **Alexis Rutschmann:** Data curation (supporting); investigation (equal); methodology (equal); writing – review and editing (equal). **Donald B. Miles:** Investigation (equal); methodology (equal); supervision (equal); validation (equal); writing – review and editing (equal). **Murielle Richard:** Data curation (equal); investigation (equal); methodology (equal); project administration (equal); supervision (equal); validation (equal); writing – review and editing (equal). **Jean Clobert:** Funding acquisition (equal); investigation (equal); methodology (equal); supervision (equal); validation (equal); writing – original draft (equal); writing – review and editing (equal).

## CONFLICT OF INTEREST STATEMENT

The authors declare that they have no competing interests.

## Supporting information


Figures S1–S6

## Data Availability

All data that support this study have been uploaded to the Dryad repository at: https://datadryad.org/stash/share/7dx4TLhUsUuDh8uxQponx13YbrG5Fil4oFkb9i8wOIM.
