# Supplementary material for: Sex‐ and state‐dependent covariation of risk‐averse and escape behavior in a widespread lizard
Source: Ecol Evol. 2023 Dec 11;13(12):e10723. doi: 10.1002/ece3.10723 (PMC10711521; doi:10.1002/ece3.10723)
Supplement: Supplementary file 1 — Figures S1–S6 [file ECE3-13-e10723-s001.docx]

**Supplementary material**

Belonging to the article of “Sex and state-dependent covariation of risk-averse and escape behavior in a widespread lizard” by Qiang Wu et al.

**List of Supporting Figures**

**Fig. S1.** The trace of Markov chains for among-individual correlations of adults overall (pooled sex), females, males and post-gravid females, respectively.

**Fig. S2.** The trace of Markov chains for among -individual correlations of gravid females under parasitic infections.

**Fig. S3.** The trace of Markov chains for among -individual correlations of males under parasitic infections.

**Fig. S4.** The trace of Markov chains for among -individual correlations of offspring overall (pooled sex) and separated by sex.

**Fig. S5.** The repeatability of escape and risk-averse behavior for adults and neonates.

**Fig. S6.** The difference of repeatability (lnRep), among-individual variance (lnVRa), and within-individual variance (lnVRw) between different sexes of juveniles for risk-taking and sprint behavior.

**Figures**

Note: the different color of chains indicate different priors as follows:

**#1 chains in black color** (uninformative prior used in the manuscript)

prior.bivar1 <- list(R=list(V=diag(2),nu=1.002), G=list(G1=list(V=diag(2), nu=1.002),

G2 = list(V = 1, nu = 0.002)))

**#2 chains in red color**

prior.bivar2 <- list(R=list(V=diag(2), nu=2),

G=list(G1=list(V=diag(2),nu=2,alpha.mu=c(0,0),alpha.V=diag(2)*1000), G2=list(V = 1, nu = 0.002)))

**#3 chains in green color**

prior.bivar3 <- list(R=list(V=diag(2),nu=2),

G=list(G1=list(V=diag(2),nu=2), G2=list(V=1,nu=2)))

**
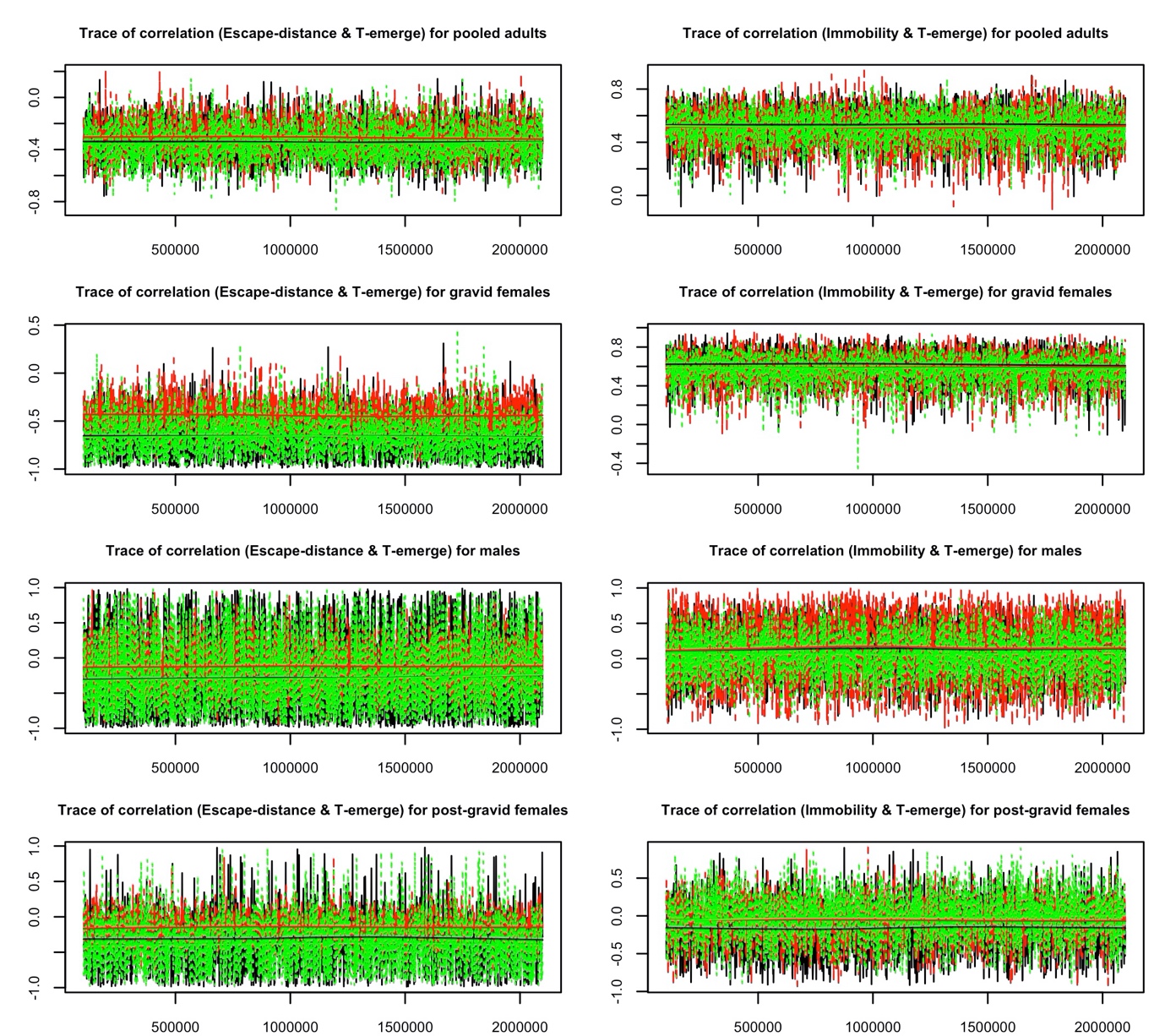
Fig. S1**. The trace of Markov chains for among-individual correlations for adults overall (pooled sex), females, males and post-gravid females, respectively. The different color of chains corresponds to respective priors to set MCMC models. Note, the chains run longer for males to achieve better convergence because of the small sample size (n=22).

**
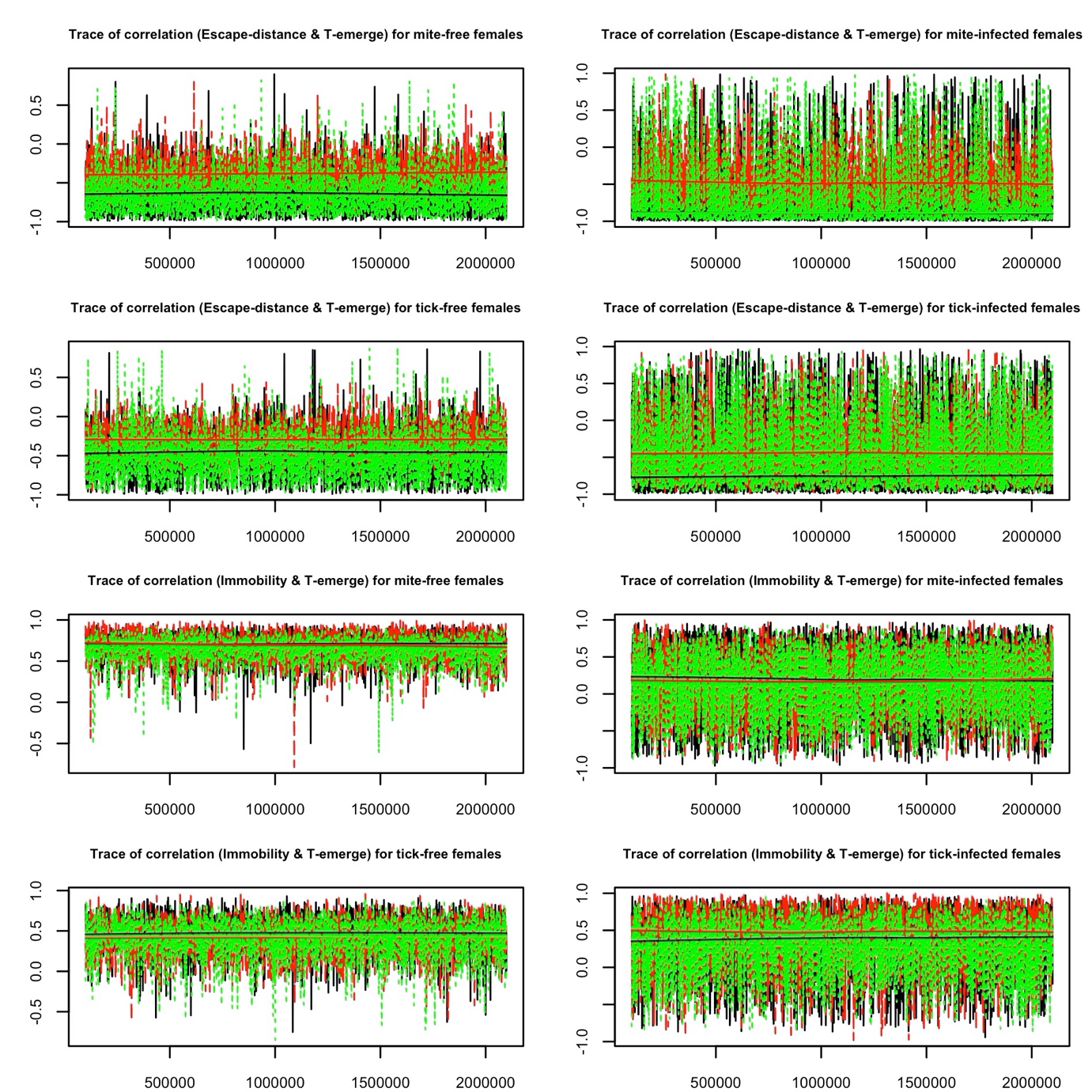
Fig. S2**. The trace of Markov chains for among-individual correlations of gravid females under parasite infections. The plots on left panel are parasite-free individuals, and on the right panel are individuals under parasite infection, respectively. The different color of chains indicates that we used different priors to set the MCMC model.

**
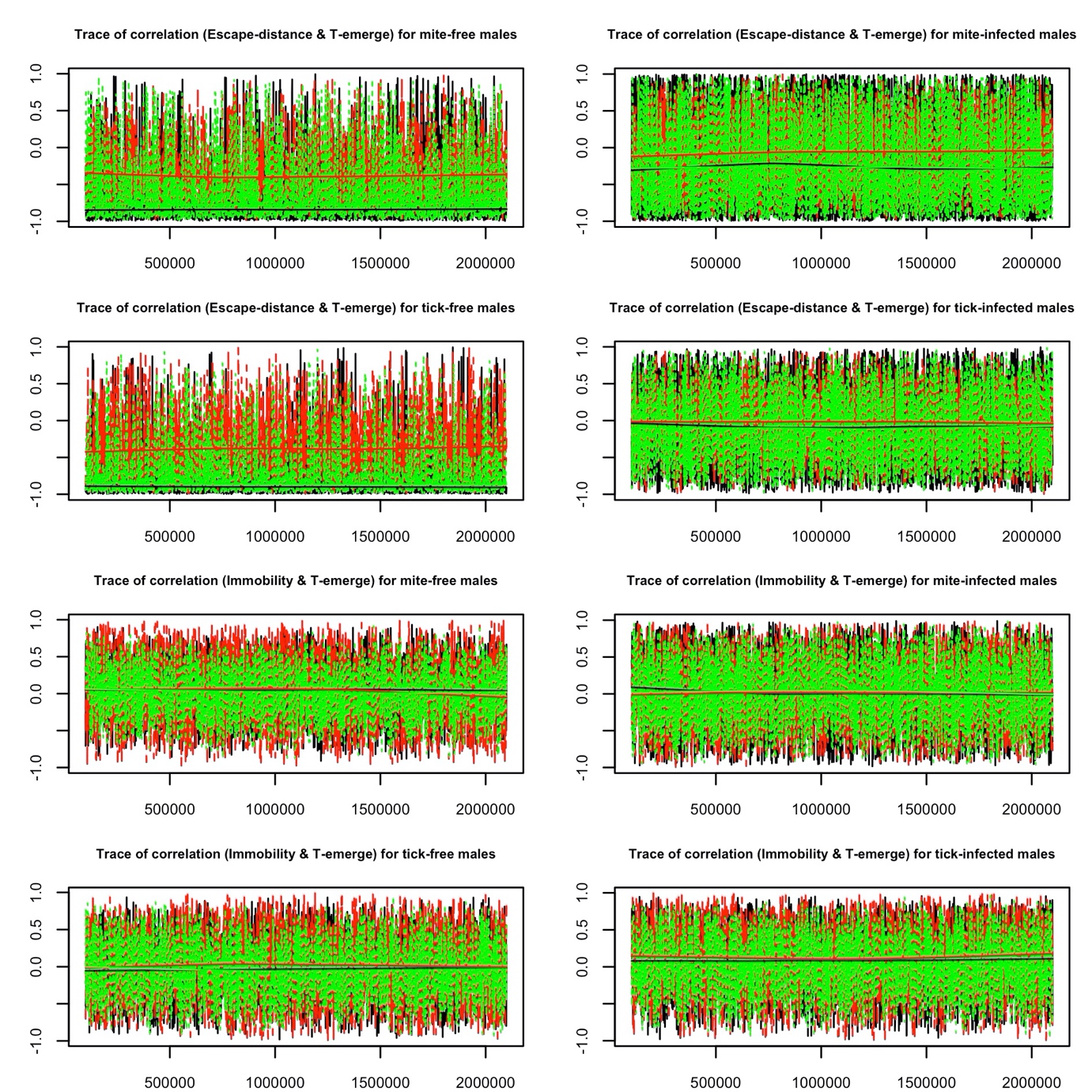
**

**Fig. S3**. The trace of Markov chains for among-individual correlations of males under parasite infections. The plots on left panel are parasite-free individuals, and on the right panel are individuals under parasite infection, respectively. The different color of chains indicates that we used different priors to set the MCMC model.

**
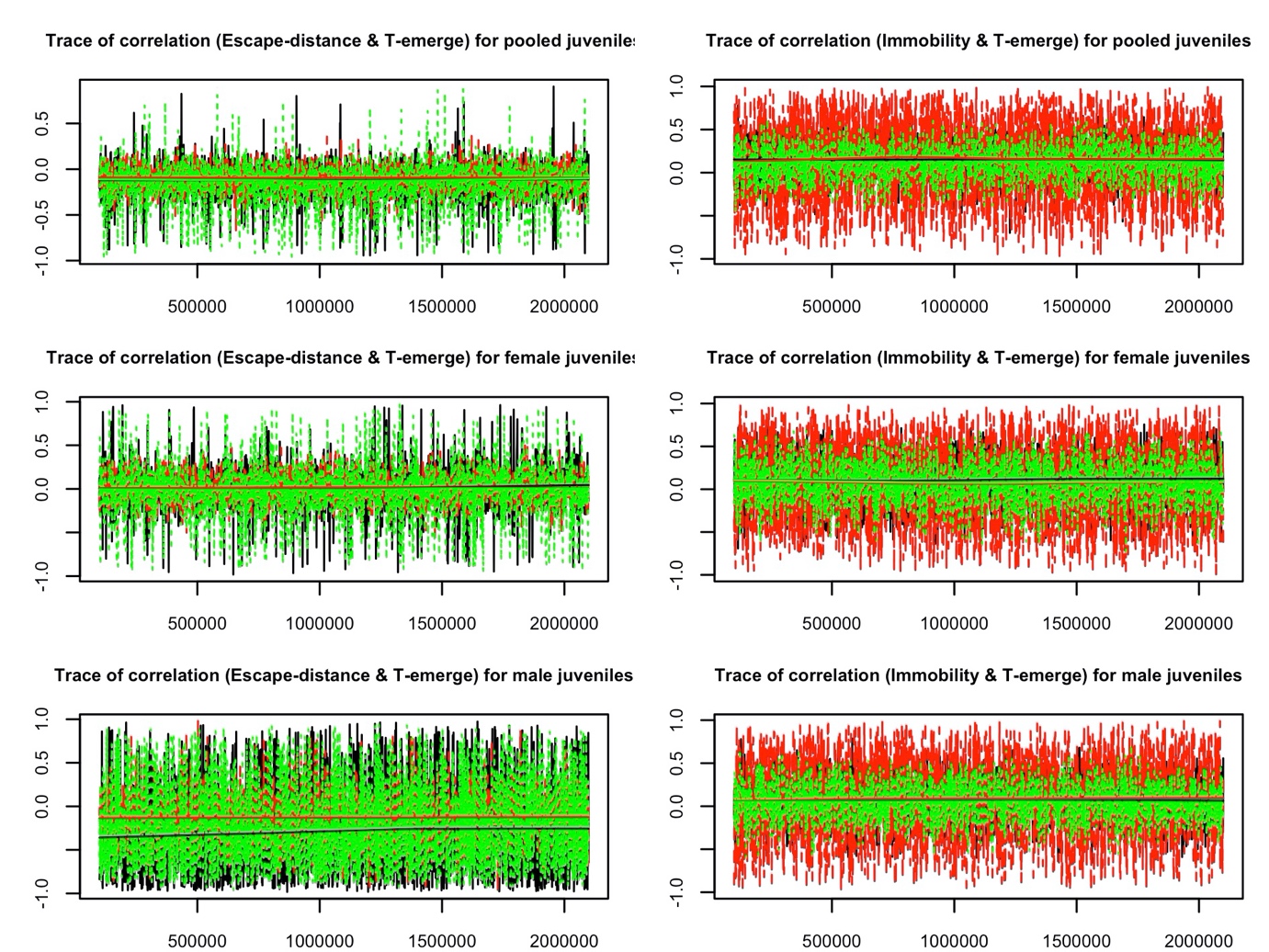
Fig. S4**. The trace of Markov chains for among-individual correlations of offspring overall (pooled sex) and separated by sex, respectively. The upper panel is the trace of correlation for pooled individuals, while the middle panel for female offspring and the lower panel for male offspring, respectively. The different color of chains indicates that we used different priors to set the MCMC model.


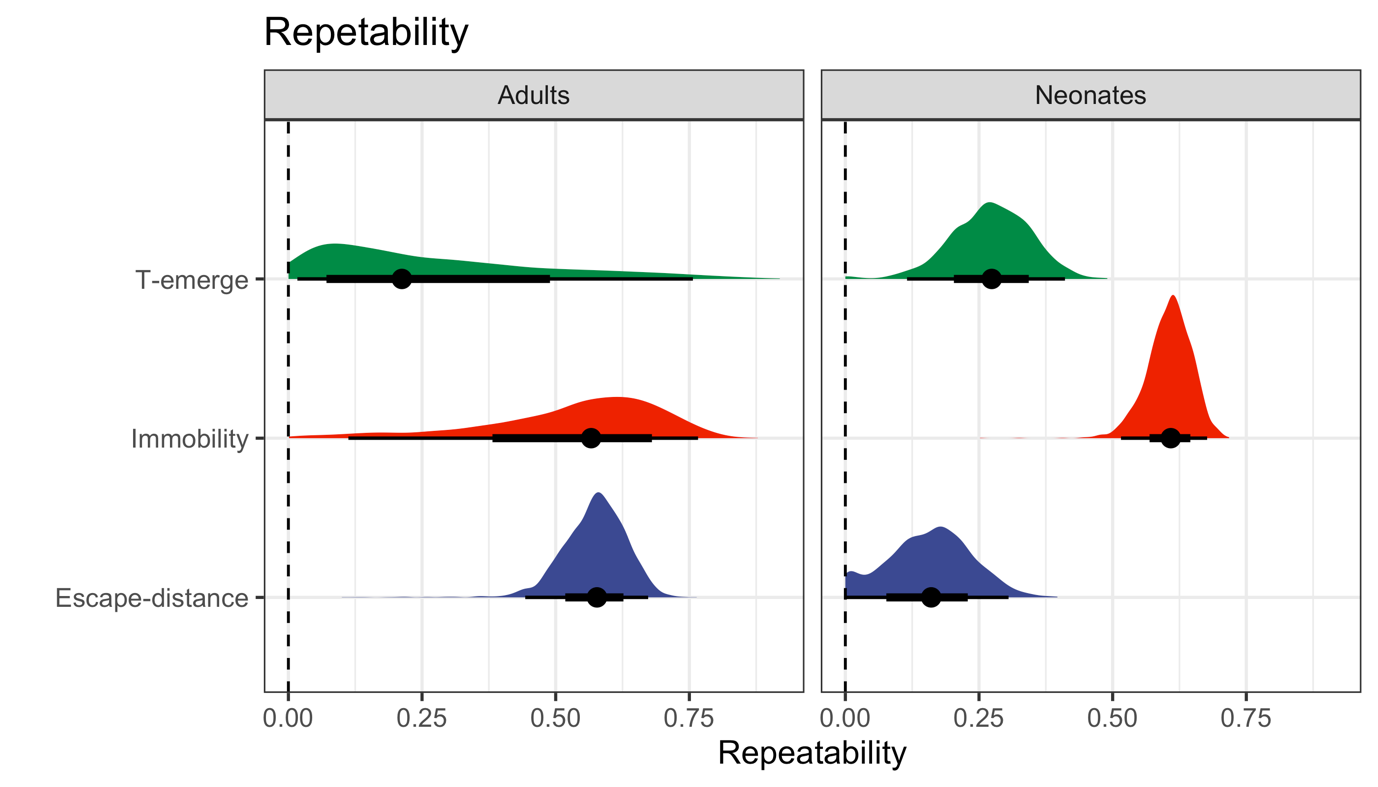


**Fig. S5.** The repeatability of escape and risk-averse behavior for adults and neonates overall.


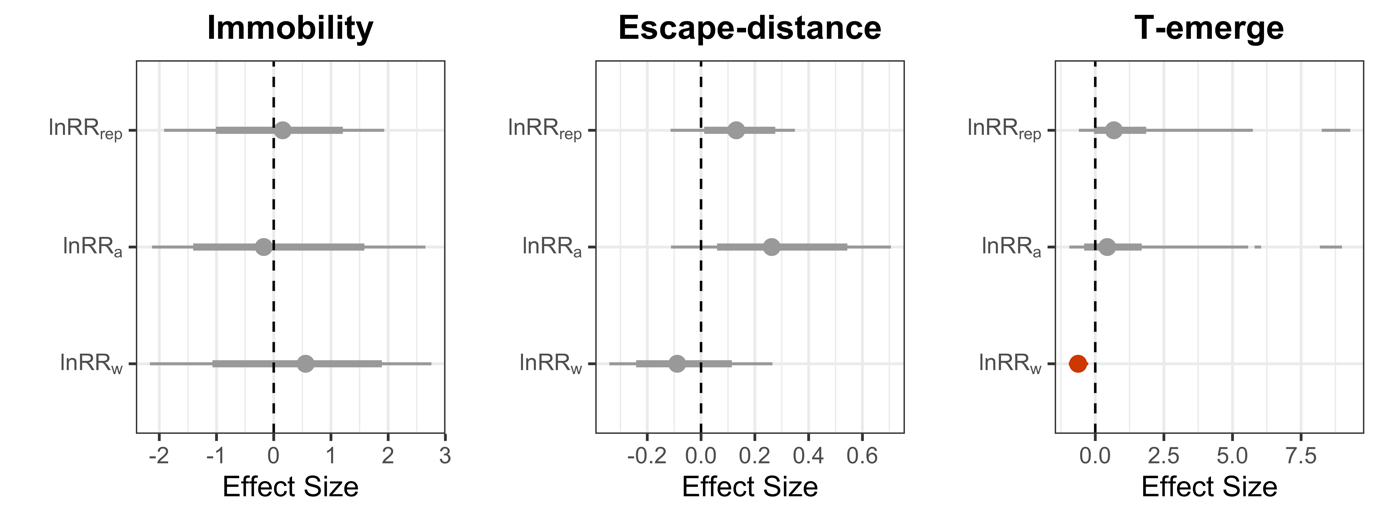


**Fig. S6.** The difference of repeatability (lnRRrep), among-individual variance (lnRRa), and within-individual variance (lnRRw) between different sexes of juveniles for two traits of the sprint behavior and one trait for the risk-taking. The magnitude of difference (effect size) is represented by the log scale ratio of repeatability/variance in one group divided by another, ln(V_1_/V_2_).
